# Pseudomyxoma Peritonei: A Case Report of a 62-Year-Old Female With Unexplained Abdominal Enlargement and Appendiceal Origin

**DOI:** 10.7759/cureus.22255

**Published:** 2022-02-15

**Authors:** Ahmed Mohfouz, Manar Hamed, Anshul Patel, Shams Tasnim, Princy Shah

**Affiliations:** 1 Internal Medicine, The Wright Center for Graduate Medical Education, Scranton, USA; 2 Oncology, Mansoura University, Faculty of Medicine, Mansoura, EGY

**Keywords:** chemotherapy response, adenomucinosis, hipec, omental cake, appendiceal, pseudomyxoma peritonei

## Abstract

Pseudomyxoma peritonei (PMP) is any mucin-producing tumor in the abdomen. If left untreated, it can compress vital abdominal organs. We describe a misleading presentation of disseminated peritoneal adenomucinosis (DPAM) of appendiceal origin. Treatment strategies for PMP vary from watchful waiting to cytoreductive surgery with hyperthermic intraperitoneal chemotherapy (HIPEC) or early postoperative intraperitoneal chemotherapy (EPIC). The rarity of PMP has precluded any randomized studies, and few institutions see sufficient cases to report a series. The purpose of this case report is to contribute to reaching a common consensus on the guidelines of pseudomyxoma peritonei. Recent studies support that cytoreduction followed by HIPEC improves survival in patients with peritoneal carcinomatosis of colorectal origin. Our case report introduced a patient with low-grade appendiceal mucinous neoplasm with visceral peritoneal penetration who underwent surgical debulking with promising results that supports the practice. Our patient has also responded well to adjuvant systemic chemotherapy without hyperthermic intraperitoneal chemotherapy.

## Introduction

Pseudomyxoma peritonei (PMP) is an uncommon disease known for mucin secreting cells seeding the peritoneum, which if left untreated, can compress vital abdominal organs [1]. The term PMP was first introduced by Werth in 1884 and despite the current incidence of over a million cases annually, the clinical syndrome of PMP remains an enigma [2]. PMP is more common in women (male: female ratio is 9:11), at a mean incidence age of 53 years [3-5]. It presents as an unexplained increase in abdominal girth (40%) [2,3], bilateral or unilateral ovarian tumors (20%), hernia sac tumors (20%), appendicitis-like syndrome (10%), and infertility (10%) [3]. Narrowing of the gastrointestinal tract occurs frequently at the parts attached to the retroperitoneum and are relatively motionless: pyloric antrum, the ileocecal valve, and the pouch of Douglas [3,4]. Unlike bowel entrapment from abdominal surgeries, PMP rarely presents as complete bowel obstruction [3-5]. A considerable majority of males present with a primary lesion in the appendix, and less commonly colorectum, gallbladder, pancreas, urachus, bladder, breast, and lung. Moreover, PMP is a late presentation after the primary event, ~5-30 years, often resulting in delayed diagnosis [2]. Almost 10% of patients die of PMP within 5.5 years of their initial presentation. The five-year and 10-year survival rate is 75% and 68% respectively [3-5]. Initial ultrasonography followed by a CT or MRI of the abdomen/pelvis is the diagnostic modality of choice. However, this disease is most often an incidental surgical or radiologic finding. CA 19-9 and carcinoembryonic antigen (CEA) serum tumor markers have demonstrated a prognostic benefit [2]. We present a case of PMP in a female with an interesting clinical picture, who eventually underwent exploratory laparotomy with tumor debulking followed by hyperthermic intraperitoneal chemotherapy [4-6].

## Case presentation

**Figure 1:**
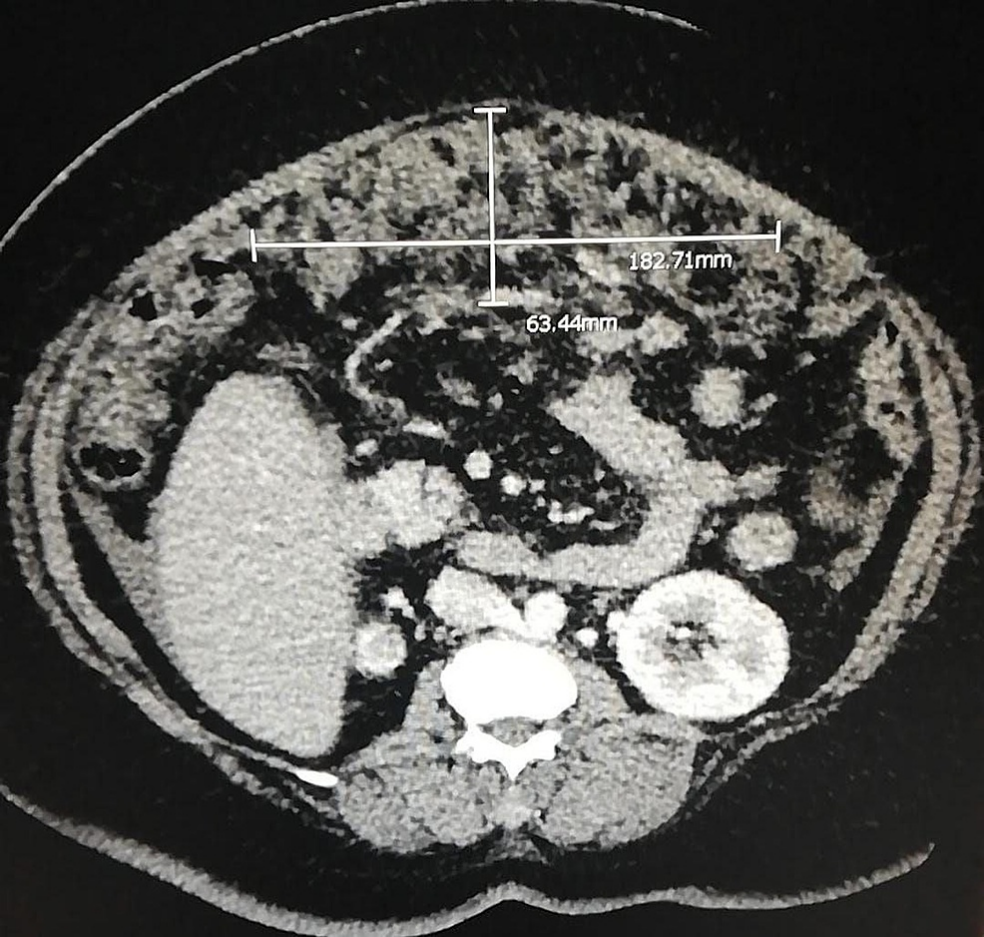
Diffuse heterogeneous enhancing soft tissue lesion is seen studded over omentum measures 182.71 X 63.44 mm describing "omental cake"

A 62-year-old female patient with a past medical history significant for type 2 diabetes mellitus, obesity, essential hypertension, and a left breast multifocal high-grade ductal carcinoma in situ (DCIS; estrogen receptor [ER]-negative, progesterone receptor [PR]-negative, strongly positive +3 score for human epidermal growth factor receptor 2 [HER2]/neu receptors) status post simple left mastectomy and sentinel lymph node (LN) biopsy with no evidence of micro-invasions and free lymph nodes, followed by six cycles of 5-fluorouracil, doxorubicin, and cyclophosphamide. During her follow-up, she complained of abdominal enlargement and generalized tenderness. Abdominal ultrasonography reported minimal ascites, enlarged early cirrhotic liver, and enlarged spleen. An MRI of the abdomen was significant for nodular stranding of the omentum, nodular and sheet-like lesions with an abnormal signal intensity related to the posterior aspect of the anterior abdominal wall (maximum thickness of 4 cm), suggesting omental caking (Figure 1). Serological analysis showed CA125 at 31, CA15-3 at 24, CEA at 72, CA19-9 at 48. Fine needle aspiration and microscopy of the omental mass showed mucinous pools with linings of mucin secreting epithelium with mild atypia. Immunohistochemical staining was positive for CK7 and SATB2 with nuclear atypia. CK20, CDX2, and mammaglobin were negative. Colonoscopy and esophagogastroduodenoscopy showed no evidence of malignant colonic or appendiceal neoplasm with the gross examination. Mammography excluded breast malignancy. She then underwent surgical debulking without hyperthermic intraperitoneal chemotherapy (HIPEC) and had a hemicolectomy with omentectomy and bilateral salpingo-oophorectomy with peritoneal stripping. The diagnosis was made after the surgical pathology report showed a low-grade appendiceal mucinous neoplasm with visceral peritoneal penetration (LAMN pT4a). Post-operative levels of CA19-9 trended down to 34, CEA trended down to 6, CA125 trended down to 26. Adjuvant chemotherapy with capecitabine with three cycles every three weeks planned over six months for a total of eight cycles. The patient continues close follow-up with oncology.

## Discussion

PMP refers to a clinical syndrome of diffuse mucinous peritoneal involvement, often associated with a primary appendiceal lesion, but includes peritoneal dissemination of a wide variety of mucin-producing invasive adenocarcinomas of the appendix, large and small bowel, lung, breast, pancreas, stomach, bile ducts, gallbladder, and fallopian tubes/ovary. PMP is not a histologically based term and is not used for staging or histologic classification of appendiceal neoplasms [2-4]. A definitive diagnosis of PMP can be made by identifying mucinous neoplastic cells/epithelium and diffuse intra-abdominal mucin. Cases without epithelium are regarded as mucinous ascites [3-5]. Some authors also require the presence of diffuse mucinous implants for a diagnosis [3]. More recently, the Peritoneal Surface Oncology Group International (PSOGI) pathologic classification for PMP has introduced a different terminology to classify PMP for the purposes of treatment selection: low-grade and acellular mucin PMP (which correspond to a ruptured LAMB and mucocele, respectively), and high-grade and signet ring cell PMP (which would correspond to peritoneal mucinous carcinomatosis) [5-7]. Histologic classification of pseudomyxoma peritonei [1] is presented in Table 1. 

**Table 1:** Histologic classification of pseudomyxoma peritonei [1]

Pathologic lesion	Criteria
Acellular mucin	Mucin within the peritoneal cavity without neoplastic epithelial cells
Low-grade mucinous carcinoma peritonei (DPAM) - "our patient"	Epithelial component typically scanty, minimal cytologic atypia, strips gland-like structures, or small cell clusters
High-grade mucinous carcinoma peritonei (PMCA)	Relatively more cellular, cribriform growth pattern, high-grade cytologic atypia, numerous mitoses
High-grade mucinous carcinoma peritonei with signet rings cells (PMCA-S)	Any lesion with a signet ring cell component, that is, round cells with intracytoplasmic mucin pushing the nucleus against the cell membrane

Appendiceal adenocarcinomas have three separate histologic types [4,5]. Mucinous type is the most common and produces abundant mucin [3,4]. The less common intestinal or colonic type closely mimics adenocarcinomas of the colon [2]. The least common, signet ring cell adenocarcinoma, is quite virulent and has a poor prognosis [2,3]. Cases with ovarian involvement are mostly found to have metastasized from an appendiceal source or another gastrointestinal source. This is why it has become a standard procedure in many institutions to perform an appendectomy routinely during the staging of ovarian neoplasms [4,5]. Mucinous peritoneal carcinomatosis may arise from other sites, but these tumors usually have signet ring histology. They may show redistribution but do not spare the small bowel and will implant and grow in the abdominal cavity with extensive small bowel involvement, resulting in a much poorer prognosis [3,4]. PMP has multiple clinical manifestations and presentations, making it a great diagnostic challenge. Patients usually experience serious health deterioration before the diagnosis is made [6,7]. The main symptom of our patient was the abdominal enlargement. Patients may also present with ascites, abdominal mass, or generalized abdominal pain. In almost 20% of cases, the evidence of PMP is an incidental surgical or radiological finding [3,4]. There are no specific guidelines from either the National Comprehensive Cancer Network (NCCN) or the European Society for Medical Oncology (ESMO) as to the appropriate staging workup for pseudomyxoma peritonei. Guidelines from the American Society of Colon and Rectal Surgeons (ASCRS) recommend the same staging workup as for colon cancer, including computed tomography (CT) of the chest, abdomen, and pelvis, and a full colonoscopy to exclude synchronous malignancies is recommended to detect the primary source of neoplasia [6,7]. A variety of serum markers like CEA, CA 19-9, and CA-125 are associated with PMP [2]. CEA is a useful prognostic marker at the time of diagnosis and for post-treatment surveillance [2,3]. Treatment strategies for PMP vary from watchful waiting to cytoreductive surgery with HIPEC or early postoperative intraperitoneal chemotherapy (EPIC) [6,7]. Recent studies support cytoreduction with peritonectomy plus HIPEC as a safe procedure suggesting improved survival rates, even in aggressive cases [7]. 

## Conclusions

Peritoneal mucinous carcinomatosis (PMCA), due to its rarity and often delayed presentation, is commonly misdiagnosed at first. There is no consensus regarding the proper management of aggressive cases. Low incidence of this disease has precluded the performance of randomized studies, and few institutions see sufficient numbers to report a series of homogeneously treated patients. Treatment recommendations and guidelines from expert groups remain wanting. A role for adjuvant chemotherapy for adenocarcinoma of the appendix has not been definitively established. Cytoreductive surgery in combination with hyperthermic intraperitoneal chemotherapy could be appropriate for aggressive PMCA.
